# Forecast of Aging of PEMFCs Based on CEEMD-VMD and Triple Echo State Network

**DOI:** 10.3390/s25133868

**Published:** 2025-06-21

**Authors:** Jie Sun, Shiyuan Pan, Qi Yang, Yiming Wang, Lei Qin, Wang Han, Ruixiang Wang, Lei Gong, Dongdong Zhao, Zhiguang Hua

**Affiliations:** 1 School of Automation, Northwestern Polytechnical University, Xi’an 710072, China; jiesun_2025@163.com (J.S.); panshiyuan@mail.nwpu.edu.cn (S.P.); yan9qi97@163.com (Q.Y.); ymwang@mail.nwpu.edu.cn (Y.W.); qinlei@mail.nwpu.edu.cn (L.Q.); hanwangaixuexi@mail.nwpu.edu.cn (W.H.); wangruixiang@mail.nwpu.edu.cn (R.W.); 2 School of Electrical and Control Engineering, Shaanxi University of Science and Technology, Xi’an 710021, China; gong_lei@sust.edu.cn

**Keywords:** fuel cell, variational mode decomposition, forecast, echo state network, remaining useful life

## Abstract

Accurately forecasting the degradation trajectory of proton exchange membrane fuel cells (PEMFCs) across a spectrum of operational scenarios is indispensable for effective maintenance scheduling and robust health surveillance. However, this task is highly intricate due to the fluctuating nature of dynamic operating conditions and the limitations inherent in short-term forecasting techniques, which collectively pose significant challenges to achieving reliable predictions. To enhance the accuracy of PEMFC degradation forecasting, this research proposes an integrated approach that combines the complete ensemble empirical mode decomposition with the variational mode decomposition (CEEMD-VMD) and triple echo state network (TriESN) to predict the deterioration process precisely. Decomposition can filter out high-frequency noise and retain low-frequency degradation information effectively. Among data-driven methods, the echo state network (ESN) is capable of estimating the degradation performance of PEMFCs. To tackle the problem of low prediction accuracy, this study proposes a novel TriESN that builds upon the classical ESN. The proposed enhancement method seeks to refine the ESN architecture by reducing the impact of surrounding neurons and sub-reservoirs on active neurons, thus realizing partial decoupling of the ESN. On this basis of decoupling, the method takes into account the multi-timescale aging characteristics of PEMFCs to achieve precise prediction of remaining useful life. Overall, combining CEEMD-VMD with the TriESN strengthens feature depiction, fosters sparsity, diminishes the likelihood of overfitting, and augments the network’s capacity for generalization. It has been shown that the TriESN markedly improved the accuracy of long-term PEMFC degradation predictions in three different dynamic contexts.

## 1. Introduction

Under the dual imperatives of escalating global energy security concerns and carbon neutrality commitments, the industrial sector has prioritized clean energy technology innovation as a strategic focus in the low-carbon transition [1]. Proton exchange membrane fuel cells (PEMFCs), recognized as next-generation power systems for transportation electrification, demonstrate exceptional potential through zero-emission operation, high energy density, and rapid start-up capabilities [2]. However, their commercial deployment remains constrained by two critical technological barriers: insufficient durability and prohibitive costs [3]. Current PEMFC stack performance exhibits a substantial gap from industrial benchmarks, with durability limited to 8000 operational hours and system costs exceeding United States Dollars (USD) 40/kW thresholds as specified in the United States Department of Energy technical roadmap [4]. Therefore, an in-depth investigation into PEMFC degradation mechanisms and an accurate forecast of degradation and remaining useful life (RUL) constitute a critical pathway to overcome technological barriers and accelerate commercialization [5].

During the operation of a PEMFC, the proton exchange membrane will age due to hydration changes, mechanical stress, and chemical erosion, and the proton conductivity will decrease. The platinum particles of the catalyst are easily dissolved and agglomerated, and the active sites are reduced. The pores of the gas diffusion layer may be blocked, affecting gas transport. The electrode support material may also be corroded. These degradation processes will reduce battery performance and life. Lifetime forecast approaches can be broadly categorized into model-based, data-driven, and hybrid methods [6]. Model-based methods, which rely on the formulation of degradation mechanisms, offer insights into internal aging processes but are constrained by the accuracy of the underlying physical models [7]. The degradation of the membrane electrode assembly (MEA) mainly includes the performance degradation of the catalyst, gas diffusion layer, and polymer electrolyte [8]. The degradation of the catalyst is mainly manifested by the dissolution and agglomeration of platinum particles and the reduction of electrochemically active surface area, which directly affects the catalytic efficiency of the PEMFC. The degradation of the gas diffusion layer may be caused by pore blockage and carbon fiber corrosion, which in turn affects the transmission efficiency of the reaction gas. In addition, the chemical and mechanical stability of the polymer electrolytes will be challenged under high potential and low humidity conditions, resulting in a decrease in proton conductivity [9]. These degradation processes are interrelated and jointly determine the overall life of PEMFCs. Therefore, a comprehensive analysis of the degradation characteristics of MEA components is crucial to accurately predict the life of PEMFCs. In [10], a semi-empirical model was developed to correlate the output voltage of fuel cells with proton exchange membrane (PEM) thickness and current density, employing an inverse fitting methodology to quantitatively characterize the temporal evolution of PEM thickness. Data-driven methods leverage the capability of machine learning to capture high-order nonlinear relationships from historical aging data, enabling precise state-of-health predictions without requiring explicit knowledge of internal degradation mechanisms [11]. In [12], ensemble empirical mode decomposition was utilized to decompose voltage time series on multiple timescales, combined with convolutional neural networks and long short-term memory networks (LSTM) to achieve accurate fuel cell life prediction. Hybrid prognostic methods integrate the strengths of both paradigms, combining the interpretability of model-based frameworks with the predictive accuracy and generalization capabilities of data-driven techniques [13]. A notable example is the hybrid method combining least squares support vector machines with enhanced regularized particle filtering for probabilistic RUL prediction [14].

Although the existing research has made progress in PEMFC multi-timescale condition monitoring, there are still three key limitations. Firstly, there is the problem of insufficient timescale decoupling. In [15], a hybrid method combining empirical modal decomposition (EMD) with LSTM was utilized to directly fuse the characteristics of different timescales. However, the physical coupling mechanism between the electrochemical process and the water management is ignored, resulting in feature confusion. Secondly, there are still online adaptive defects in PEMFC multi-timescale condition monitoring. Traditional signal decomposition methods, such as VMD [16], need to preset the number of modes, which is difficult to adapt to the change of feature dimension under the dynamic conditions of PEMFCs. Thirdly, most methods predict that the aging trend of PEMFCs is in the interpretable bottleneck stage at present. Existing deep learning models, such as temporal convolutional networks (TCNs), have improved accuracy but cannot establish an explicit correlation between modal components and internal mechanisms such as physical degradation, including catalyst dissolution [17].

The echo state network (ESN) demonstrates significant advantages in fuel cell lifetime prediction through its unique reservoir computing paradigm. Its randomly sparsely connected reservoir structure efficiently captures nonlinear dynamics and multivariate-coupled degradation characteristics, while the output-layer-only training mechanism eliminates back-propagation overhead, enabling rapid deployment and online updates. The leaky integrator and multi-scale architecture further enhance its capability to simultaneously resolve fast-varying and slow-varying degradation patterns, making it a robust tool for real-time prognostics in transportation electrification systems. Li et al. [18] proposed an ensemble ESN architecture utilizing virtual steady-state superimposed voltage as a health indicator (HI) for long-term RUL prediction under both steady-state and dynamic operating conditions. In [19], a multi-reservoir echo state network with minor reservoirs was proposed for PEMFC degradation prediction, employing particle swarm optimization (PSO) to determine optimal reservoir configuration parameters, including main reservoir quantity and neuron population. Hua et al. [20] developed a data-driven approach combining a discrete wavelet transform, ESN, and genetic algorithm to improve the accuracy of forecasting the RUL through multi-resolution feature extraction. In [21], a hybrid framework integrating a bidirectional LSTM, bidirectional gated recurrent unit, and ESN demonstrated improved short-term degradation prediction accuracy under limited training datasets. Additionally, Jin et al. [22] proposed a cyclic reservoir with jumping architecture, which transfers reservoir state information through cyclic jumps, significantly enhancing stack voltage prediction and RUL estimation. For dynamic load applications, Mezzi et al. [23] integrated an ESN with Markov chains to predict fuel cell RUL without prior load distribution knowledge, eliminating dependence on future load profile assumptions. The evolution from basic ESN architectures to hybrid frameworks highlights reservoir computing’s pivotal role in addressing PEMFC degradation’s temporal complexity and operational variability. Integrating optimization algorithms and multi-scale signal processing has advanced predictive maintenance strategies for transportation electrification [24].

To address the issue of multi-timescale factors interfering with prediction accuracy during the aging process of PEMFCs, this paper proposes a complete ensemble empirical mode decomposition with variational mode decomposition (CEEMD-VMD)-based triple echo state network (TriESN) network structure to achieve precise RUL prediction. First, the relative power-loss rate (RPLR) aging data is smoothed and reconstructed using a least mean square (LMS) adaptive filter. Subsequently, the processed training data is decomposed using CEEMD, and the high-frequency RPLR signals are discarded. High-frequency and low-frequency RPLR signals are extracted from the remaining signals and used as inputs. Next, VMD is employed to further subdivide the low-frequency signals to enhance decomposition accuracy. An improved TriESN structure model is then utilized to predict the RUL of the PEMFC, with parameters such as leakage rate, spectral radius, regularization factor, and output weights optimized using PSO. Finally, the proposed method is verified under three different dynamic conditions and compared with typical data-driven methods currently in use, demonstrating its feasibility and effectiveness in the field of PEMFC life prediction.

## 2. Experimental Setup and Methodology

### 2.1. Experimental Setup

**Figure 1:**
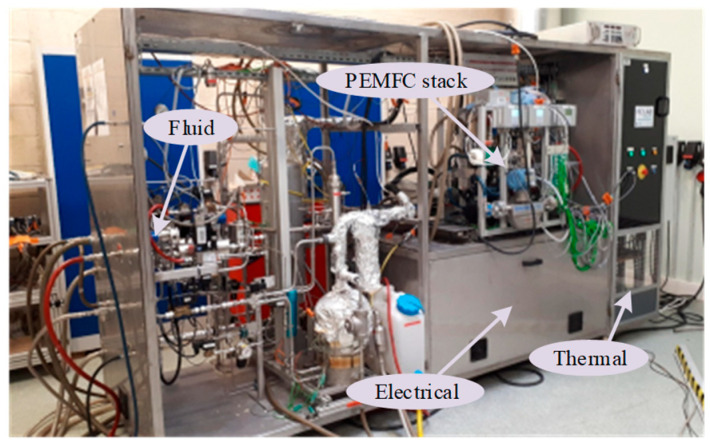
The experimental configuration of the PEMFC system.

The deterioration dataset sourced from the Fuel Cell Laboratory (FCLAB) [25], encompassing performance degradation under diverse current profiles, is utilized to validate the proposed lifetime prediction framework. The PEMFC system’s operational states and parameter configurations are systematically recorded under three distinct conditions: fully dynamic 1 (experiment 1), fully dynamic 2 (experiment 2), and fully dynamic 3 (experiment 3). These are from the micro-combined heat and power (μ-CHP). For the test, the stack consists of eight cells operating at 80 °C with an effective area of 220 cm^2^. The test bench has an electrical power output of 1.0 kW. In this task, a subset of the standard specifications and operational parameters are considered. These include the physical dimensions of the system, which are 220 mm × 160 mm × 186 mm. The anode/cathode stoichiometry ratio is 1.5/2, and the inlet pressure for both the anode and cathode is specified at 150 kPa. The cooling flow rate is 2 L/min, and the pressure drop across the system is 30 kPa. The 405 h test is divided into four stages. The variation of load current at each stage includes the stage 1, the stage 2, the stage 3, and the stage 4. Concretely, the total duration of stage 1 is 0–125 h, of which the current density is 0.36 A/cm^2^ at 0–25 h, and the current density is 0.45 A/cm^2^ at 25–125 h. The total duration of stage 2 is 125–250 h, of which the current density is 0.45 A/cm^2^ to 0.23 A/cm^2^ at 125–225 h, and the current density is 0.23 A/cm^2^ at 225–250 h. The total duration of stage 3 is 250–375 h, and the current density is 0 A/cm^2^ to 0.23 A/cm^2^. The total duration of stage 4 is 375–405 h, and the current density is 0.23 A/cm^2^. Terminal RPLR is adopted as the primary health indicator, enabling the enhanced model to perform multi-step predictions across varied operational scenarios. The experimental configuration of the PEMFC system is illustrated in Figure 1. The dynamic testing experiment is conducted using a PEMFC stack from a micro-combined heat and power (*μ*-CHP) system for aging tests. The electrical power of the PEMFC stack is approximately 1 kW, and the testing procedure is similar to that of steady-state testing. All three fuel cell stacks consist of eight single cells each, with an active area of 220 cm^2^. All electrical variables (current and voltage) and operational parameters (flow rate, temperature, pressure, etc.) are measured on the experimental test bench. 

During the operation of the PEMFC system, the actual power output can be determined by multiplying the measured voltage and current values. At a certain current *I_t_*, the RPLR can be calculated by

(1) $$\mathrm{RPLR}=(P_{t}-P_{0})/P_{0}$$

where *P_t_* and *P*_0_ are the actual power and initial power at current *I_t_*. Thus, the RPLR exhibits an overall monotonic decreasing trend due to the inherent degradation mechanisms within the PEMFC system. Regarding the RPLR as the HI can weaken the effects of current because *P_t_* and *P*_0_ are at the same current level. Further, the calculation process of RPLR is simple and can be used online.

An adaptive filtering mechanism, the LMS algorithmic framework, is employed for data conditioning and reconstruction. This approach effectively retains the intrinsic degradation trend of the output RPLR while attenuating high-amplitude noise interference. Post decomposition, the network architecture parameters are optimized as follows: input weights ***W***_in_ are initialized within [−0.5, 0.5]; sub-reservoir cyclic weights ***W*** are bound by [−0.5, 0.5]; and the neuron counts across sub-reservoirs follow a uniform distribution (C_1_ = C_2_ = C_3_). The leakage rates α_1_, α_2_, and α ∈ (0,1), the regularization factors a_1_, a_2_, and a_3_, ∈ (0.001, 0.009], while the parallel output weights e_1_ and e_2_ ∈ (0.001, 0.999].

### 2.2. Implementation Plan

**Figure 2:**
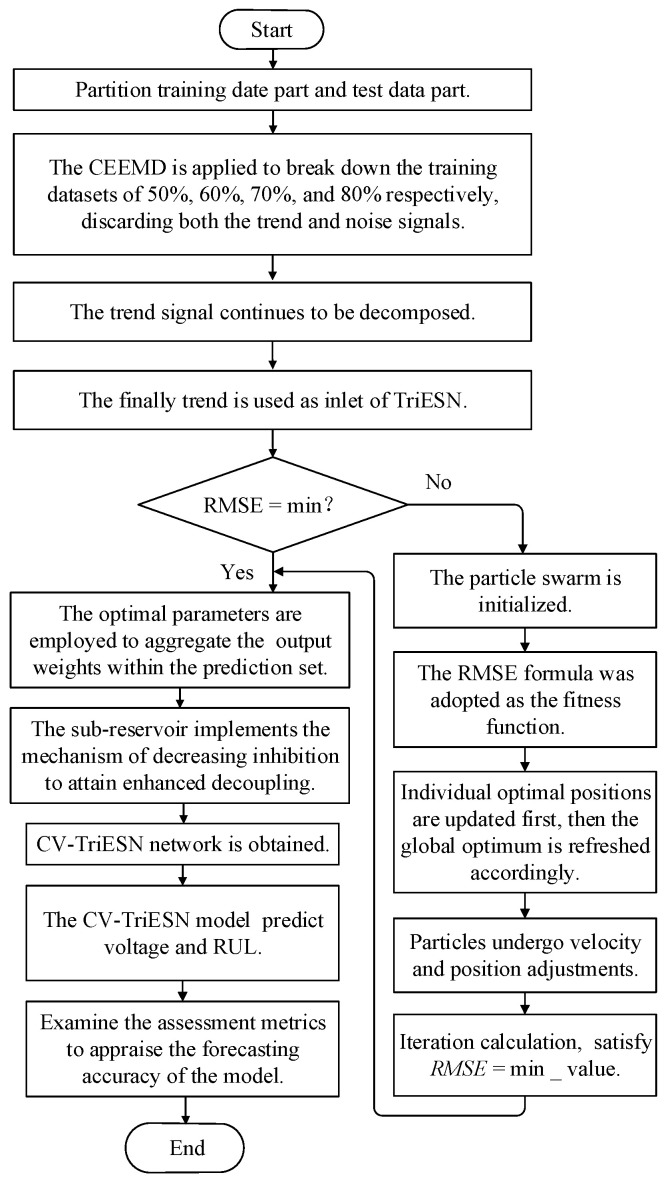
The implementation scheme of constructing the CV-TriESN.

This study presents an innovative approach for PEMFC degradation prediction by integrating CEEMD and VMD decomposition with TriESN (CV-TriESN) modeling and PSO optimization. Firstly, CEEMD decomposes PEMFC aging data into trend terms and noise components, where the former is preserved as the primary degradation indicator. At the same time, the trend terms continue to be decomposed by VMD. Then, the processed low-frequency signal serves as distinct input for the TriESN network, which is specifically designed to capture long-term degradation patterns. Finally, the PSO algorithm fine-tunes TriESN’s structural parameters to enhance prediction accuracy for both RUL and gradual wear-out. The application methodology for constructing the CV-TriESN is shown in Figure 2.

### 2.3. Evaluation Index

In this investigation, the output RPLR is adopted as the primary performance indicator. Through advanced signal processing techniques, the raw RPLR data undergo comprehensive filtration and reconstruction across multiple operational regimes, effectively suppressing noise measurements while preserving the intrinsic degradation characteristics. The model’s predictive accuracy is quantitatively assessed using two established metrics, root mean square error (RMSE) and mean absolute percentage error (MAPE), whose mathematical formulations will be elaborated in subsequent sections. This methodological approach ensures robust evaluation of the PEMFC’s performance degradation under varying working conditions.

(2) $$\mathrm{RMSE}=\sqrt{\frac{1}{Q}\overset{Q}{\underset{i=1}{\sum }}{\left(b_{i}\left(k\right)-{b_{i}}^{\prime }\left(k\right)\right)}^{2}}$$

(3) $$\mathrm{MAPE}=\frac{1}{Q}\overset{Q}{\underset{i=1}{\sum }}\left|\frac{b_{i}\left(k\right)-{b_{i}}^{\prime }\left(k\right)}{b_{i}\left(k\right)}\right|$$

where *b_i_*(*k*) is the value of *i*-th forecasted RPLR; *b_i_*′(*k*) indicates the value of *i*-th measured RPLR; and *Q* stands for the total number of prediction results.

## 3. Methodology

### 3.1. Complementary Ensemble Empirical Mode Decomposition

The CEEMD is a signal separation technique developed based on EMD and ensemble empirical modal decomposition (EEMD). It aims to solve the reconstruction error problem of EEMD. The CEEMD improves the reconstruction performance of EEMD by introducing complementary noise based on EEMD [26]. The algorithmic flow of CEEMD is as follows: Firstly, two complementary white noises *n_i_*(*t*) and −*n_i_*(*t*) with the same amplitude and opposite sign are generated for the original signal *x*(*t*).

(4) $${x_{i}}^{+}(t)=x(t)+n_{i}(t),{x_{i}}^{-}(t)=x(t)-n_{i}(t)$$

Secondly, the noise is superimposed on the original signal respectively, and the EMD decomposition of the two new signals is performed to obtain the intrinsic mode function IMF. Finally, the modal components are averaged to eliminate the effect of noise.

(5) $${\mathrm{IMF}}_{j}(t)=\frac{1}{2}\left[{{\mathrm{IMF}}_{j}}^{+}(t)+{{\mathrm{IMF}}_{j}}^{-}(t)\right]$$

where IMF*_j_*(*t*) represents the final decomposition result; IMF*_j_*^+^(*t*) and IMF*_j_*^−^(*t*) represent the two sets of signals decomposed by EMD, respectively.

### 3.2. Variational Mode Decomposition

VMD transforms the signal decomposition problem into a variational optimization problem, the core of which is to represent the input signal as a finite sum of IMFs with different center frequencies, with each mode tightly concentrated around its center frequency, and to achieve effective separation by optimizing the bandwidth of each mode [27]. Through the mathematical variational framework, VMD effectively solves the modal overlapping problem existing in the EMD class of methods and significantly improves the decomposition quality. The variational constraint model obtained by constructing the initial signal *x*(*t*) is shown as

(6) $$\begin{array}{l} \underset{\left\{u_{k}\right\},\left\{\omega_{k}\right\}}{\min}\sum_{k=1}^{K}{\left\|\partial_{t}\left[\left(\delta (t)+\frac{j}{\pi t}\right)u_{k}(t)\right]e^{-j\omega_{k}t}\right\|}_{2}^{2}\\ \sum_{k=1}^{K}u_{k}(t)=x(t) \end{array}$$

where ∂(*t*) is the partial derivative operator; *δ*(*t*) is the Dirac distribution function; *u_k_*(*t*) is the *k*th mode component; ω_*k*_ is the center frequency of the *k*th mode.

To solve the constrained optimization problem of this model, the constrained variational problem is converted into an unconstrained one through the application of quadratic penalty terms and the Lagrange multiplier method, and it is ultimately resolved by employing the alternating direction method of multipliers. Aiming at the high-frequency signal processing after CEEMD-VMD decomposition, this paper proposes a quantitative discarding criterion based on the energy–frequency dual threshold. When the IMF component meets the energy ratio of less than 5% (α = 0.05) and the dominant frequency is greater than 50 Hz (beyond the effective frequency band of the PEMFC), it is eliminated. At the same time, it is required that the retained component needs to match the known fault characteristic frequency band or continue to appear greater than or equal to 5 cycles.

### 3.3. Echo State Network

**Figure 3:**
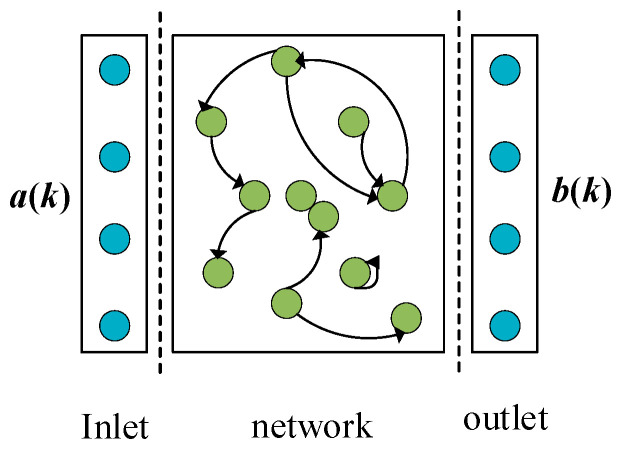
The architecture in the ESN.

The ESN represents a specialized recurrent neural network architecture designed for temporal data modeling. This framework employs a unique reservoir computing paradigm, where a sparsely connected, high-dimensional dynamical system of neurons captures the network’s memory properties [28]. As illustrated in Figure 3, the canonical architecture comprises three fundamental components: an input layer, a nonlinear transformation reservoir, and an output layer. The network’s predictive performance is principally governed by systematic optimization of reservoir parameters (including leakage rate and spectral radius) and regularization coefficients, which collectively determine the output weight computation process, with detailed implementation methodologies provided.

The ESN architecture is characterized by input feature, reservoir feature, and output feature, with the state update governed by the equation. The number of input, reservoir state, and output nodes in the ESN are *X*, *Y*, and *Z*, respectively.

(7) $$b^{\sim }\left(k\right)=f\left(A_{in}*a\left(k\right)+A*b\left(k-1\right)\right)$$

(8) $$b\left(k\right)=\left(1-\mu \right)*b\left(k-1\right)+\mu *b^{\sim }\left(k\right)$$

where *f*(·) represents the reservoir neuron activation function (typically tanh(·)); *μ* ∈ (0, 1] denotes the leakage rate; ***a***(*k* − 1) ∈ ***R****^Zx^* is the input vector; ***b***(*k*) ∈ ***R****^Z_Y_^* and ***b***^~^(k) ∈ ***R****^Z_Y_^* are the activation and updated state vectors, respectively; ***b***(*k* − 1) ∈ ***R****^Z_Y_^* is the previous reservoir state; and ***A****_in_* ∈ ***R****^Z_Y_×^^Z_X_^* and ***A*** ∈ ***R****^Z_Y_×^^Z_Y_^* are the input and recurrent weight matrices. The output update process and state equation are formulated as follows:

(9) $$\gamma^{\sim }\left(k\right)=f_{out}\left(A_{out}*\left(a\left(k-1\right),b\left(k-1\right)\right)\right)$$

(10) $$\gamma \left(k\right)=A_{out}*\left[a\left(k-1\right);b\left(k-1\right)\right]$$

where *f*_out_(·) denotes the output layer activation function; ***γ***^~^(*k*) represents the updated output state at step *k*; ***γ***(*k*) represents the updated output vector at step *k*; and ***A****_out_* ∈ ***R****^Z_Z_×(Z_X_+Z_Y_)^* is the output weight matrix.

(11) $$A_{out}=arg\;min\sqrt{\frac{1}{F}\overset{F}{\underset{n=1}{\sum }}{\left(\gamma_{n}\left(k\right)-\gamma_{n}{\left(k\right)}^{target}\right)}^{2}}$$

(12) $$A_{out}=D^{\mathrm{target}}C^{T}{\left(CC^{T}+\varepsilon E\right)}^{-1}$$

where ***F*** corresponds to the total number of data points in the training dataset; ***γ**_n_*(*k*) represents the value of *n*-th predicted RPLR value; and ***γ****_n_*(*k*)*^target^* represents the value of n-th actual RPLR value. In addition, ***C*** is the reservoir output matrix; ***D***^target^ contains target values; ***ε*** is the regularization parameter; ***E*** denotes the identity matrix; and (·)*^T^* is transpose calculation symbol.

### 3.4. Triple Echo State Network

**Figure 4:**
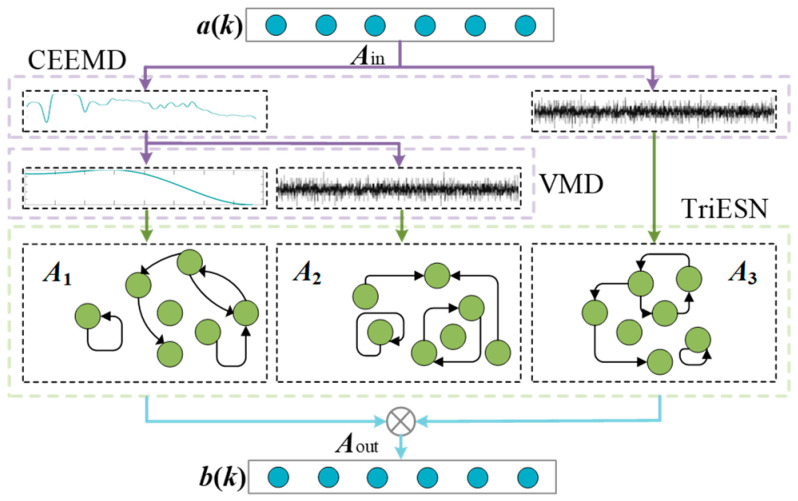
The structure of the CV-TriESN.

The TriESN architecture reconstructs the conventional single-reservoir ESN framework by employing triple parallel sub-reservoirs with sequential pairwise merging operations. This configuration processes CEEMD-VMD-derived intrinsic mode functions as inputs, with the reservoir quantity determined through a comprehensive evaluation of input-parameter compatibility and computational efficiency–prediction accuracy trade-offs. In this study, we develop an enhanced ESN variant, the CV-TriESN model incorporating five parallel sub-reservoirs. The TriESN architecture integrates CEEMD-VMD-based signal decomposition with parallel reservoir processing, while its critical parameters (including leakage rate, regularization coefficient, spectral radius, and output weights) are optimized via the PSO algorithm. Some related parameters of PSO are set, including the following: the population size is 50; the maximum number of iterations is 200; the inertia weight linearly decreases from 0.9 to 0.4; and the acceleration constant is c1 = c2 = 1.49445. To optimize the three key hyperparameters and weight distribution factors of the CV-TriESN, the spectral radius, leakage rate, regularization coefficient, and input scaling factor of the optimal sub-reservoir are found. The structure of the CV-TriESN is shown in Figure 4.

The reservoir architecture employs a uniformly partitioned configuration, where the *W* neurons are equally distributed across three distinct sub-reservoirs. Each sub-reservoir maintains parameter settings (including leakage rate, spectral radius, and regularization factor) consistent with conventional ESN design principles. The hierarchical output structure is mathematically expressed as follows:

(13) $$d_{1}\left(k\right)=A_{1out}*\left[a_{1}\left(k-1\right);b_{1}\left(k-1\right)\right]$$

(14) $$d_{2}\left(k\right)=A_{2out}*\left[a_{2}\left(k-1\right);b_{2}\left(k-1\right)\right]$$

(15) $$d_{3}\left(k\right)=A_{3out}*\left[a_{3}\left(k-1\right);b_{3}\left(k-1\right)\right]$$

(16) $$d^{*}\left(k\right)=d_{1}\left(k\right)+d_{2}\left(k\right)$$

(17) $$d\left(k\right)=d^{*}\left(k\right)+d_{3}\left(k\right)$$

In Equations (13)–(17), the mathematical formulation is characterized by ***d****_n_*(*k*) ∈ ***R****^Z_Y_^* (*k* = 1, …, 3), representing the output vector of the *n*th sub-reservoir at time step *k*, where ***A***_(*n*)*out*_ ∈ ***R****^Z_Y_×(Z_X_+Z_Y_)^* denotes the corresponding output weight matrix. The temporal dynamics are captured through ***a****_n_*(*k* − 1) ∈ ***R****^Z_X_^* as input vectors at consecutive time steps (*k* − 1), while ***b****_n_*(*k* − 1) ∈ ***R****^Z_Y_^* represents the neuron activation vectors of the *n*th sub-reservoir at these time instances. The final output is computed through a weighted summation of sub-reservoir outputs, expressed as the output synthesis process, and is mathematically characterized as follows: d*(*k*) ∈ ***R****^Z_Z_^* denotes the weighted combination of ***d***_1_(*k*) ∈ ***R****^Z_Z_^* and ***d***_2_(*k*) ∈ ***R****^Z_Z_^*. The final RPLR prediction ***d***(*k*) ∈ ***R****^Z_Z_^* is obtained through the weighted summation of d*(*k*) and ***d***_3_(*k*) ∈ ***R****^Z_Z_^*.

Compared to the ESN, the CV-TriESN model has an advantage in decoupling to improve prediction accuracy. Firstly, the diversity of dynamic response increases, and each sub-reservoir tends to capture different characteristic modes of the input signal. Secondly, through parallel processing of signal components and cross-scale coupling suppression, multi-scale feature extraction is realized to reduce the mutual interference of different frequency band features at the neuron level. Finally, by orthogonalizing the dynamic response, the redundancy of neuron output is reduced. The above characteristics of dynamic diversity, multi-scale specialization, and redundancy suppression jointly improve the modeling ability and prediction accuracy of the model for complex degradation of the PEMFC. The CV-TriESN model utilizes two key parameters—leakage rate (*μ_k_*) for balancing historical/current state influences and spectral radius (*ρ_k_*) for controlling multi-timescale dynamics—to address the PEMFC’s complex aging characteristics across millisecond-level electrochemical responses to hundred-hour material degradation. By combining CEEMD-VMD-based multi-scale decomposition with PSO-optimized parallel reservoir *ρ_k_* computing, this approach simultaneously enhances feature extraction efficiency and prediction accuracy, effectively mitigating cross-timescale interference for improved long-term RUL estimation of fuel cell systems. When deploying the CEEMD-VMD-TriESN model in an actual PEMFC system, there may be some practical obstacles. It should be considered comprehensively from the aspects of computing resources, real-time performance, data quality, and engineering adaptability. However, it needs detailed analysis according to the actual situation.

## 4. Result and Discussion

### 4.1. Under Dynamic Conditions (Experiment 1)

Due to the problem of multi-timescale interference prediction accuracy in the PEMFC aging process, these multi-timescale interferences need to be considered. Multiple timescales refer to various physical and chemical processes that occur on different timescales. These processes include sub-second electrochemical processes, minute-level driving cycles, and thousands of hours of battery aging. This multi-scale characteristic is also reflected at the data level; that is, the change of health indicators is affected by different factors at different time points, showing multi-scale characteristics. In order to accurately describe the actual operating state of a PEMFC, it is necessary to extract the deep nonlinear characteristics of aging data from a multi-scale perspective. In this paper, a CV-TriESN hybrid model is proposed to solve the problem of multi-timescale (second-level electrochemical response/minute-level water management/hour-level attenuation) interference prediction accuracy in the PEMFC aging process, and its innovative solution is reflected in three levels. At the timescale decoupling level, the CEEMD-VMD cascade decomposition separates the original signal into IMF components corresponding to the characteristic timescale. The high-frequency IMF (0.1–1 Hz) captures the electrochemical polarization transient, the intermediate frequency IMF (0.01–0.1 Hz) characterizes the water management fluctuation, and the low-frequency IMF (<0.01 Hz) reflects the long-term attenuation trend. At the scale adaptive processing level, the three sub-reservoirs of the TriESN are optimized separately. The high-frequency component is processed by the fast reservoir, the medium-speed reservoir tracks the medium-frequency change, and the slow reservoir remembers the low-frequency evolution. At the cross-scale feature fusion level, the weight ratio of each scale feature is determined by cross-validation to suppress the interference of high-frequency noise on long-term prediction and improve the prediction accuracy of PEMFC aging.

**Figure 5:**
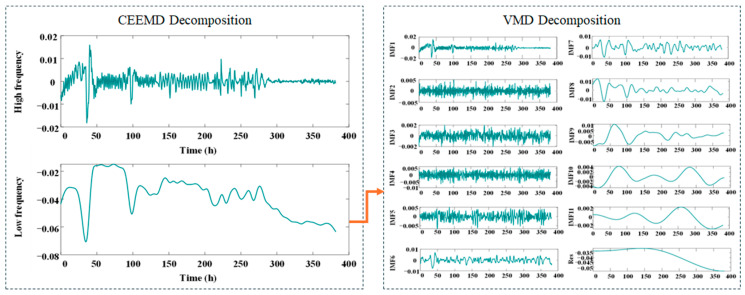
The result of decomposition under experiment 1 by CEEMD-VMD.

In experiment 1, the proportion of training is set to 50%, 62.5%, 75%, and 87.5% of the total length 382 h. Under initial experimental conditions, the 382 h aging RPLR dataset was systematically preprocessed and divided into four subsets based on temporal characteristics. To reveal multi-scale degradation features, the raw RPLR signals were adaptively decomposed using CEEMD, effectively separating high-frequency and low-frequency components. The LF components, representing long-term degradation trends, were further refined through VMD, yielding 11 IMFs and one residual component in Figure 5.

**Figure 6:**
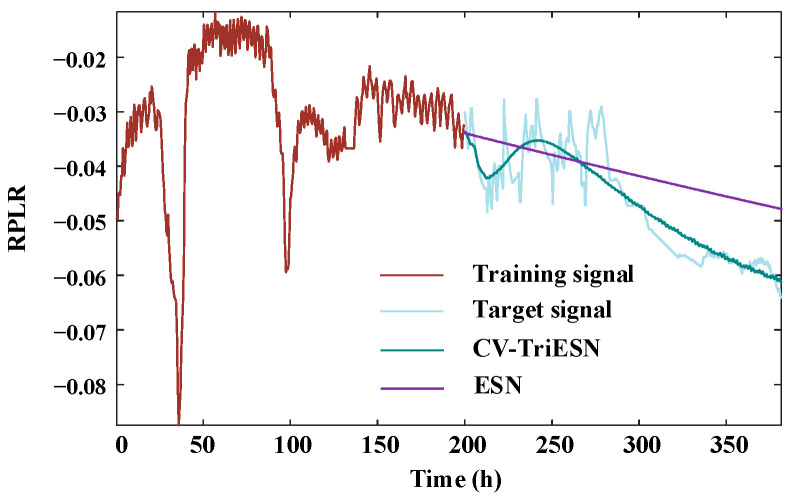
The prediction under experiment 1.

The CV-TriESN model demonstrates significantly superior performance compared to the standard ESN architecture in RPLR forecast tasks. As illustrated in Figure 6, following a 200 h training phase, the prediction curve of the CV-TriESN closely matches the target signal. In the time interval from 200 h to 350 h, its dynamic change trend accurately captures the nonlinear characteristics of RPLR degradation. In contrast, the prediction results of the traditional ESN show a clear linear bias, failing to effectively fit the complex decay pattern of actual RPLR. In summary, the deviation of the predicted values from the target values of the CV-TriESN model is significantly smaller than that of the traditional ESN method, confirming its strong robustness and generalization performance in complex time series prediction tasks.

Table 1 contrasts the prediction accuracies of the ESN, LSTM, Transformer, and CV-TriESN across varying training durations. CV-TriESN demonstrates RMSE reductions of 4.31%, 16.23%, and 11.82% at 200 h, 250 h, and 350 h, respectively, and MAPE decreases of 11.25%, 10.63%, and 21.10% compared to the ESN. This improved long-term RPLR prediction accuracy is achieved through multi-timescale feeding signals and reservoir partitioning in the CV-TriESN. The CV-TriESN showed the best performance in all the training stages, especially in the 350 h stage, and its RMSE was 0.00097, which was significantly lower than other models. In addition, the Transformer model is second in most stages, and its error is 5–15% higher than that of the CV-TriESN, while the LSTM model is 10–20% higher than that of Transformer. As a benchmark model, the ESN has the weakest performance, which verifies the improvement effect of the CV-TriESN.

### 4.2. Under Dynamic Conditions (Experiment 2)

**Figure 7:**
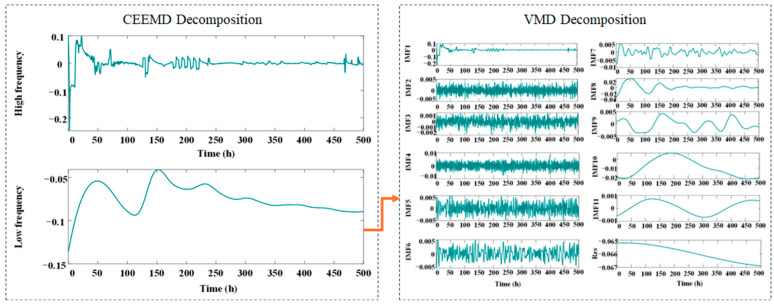
The result of decomposition under experiment 2 by CEEMD-VMD.

In experiment 2, the proportion of training is set to 40%, 50%, 60%, and 70% of the total length 500 h. For the second dataset, the preprocessed 500 h aging RPLR signals were segmented into four temporal groups. Figure 7 presents the CEEMD-VMD decomposition results of the aging RPLR signals, demonstrating the effective separation of multi-scale degradation characteristics.

**Figure 8:**
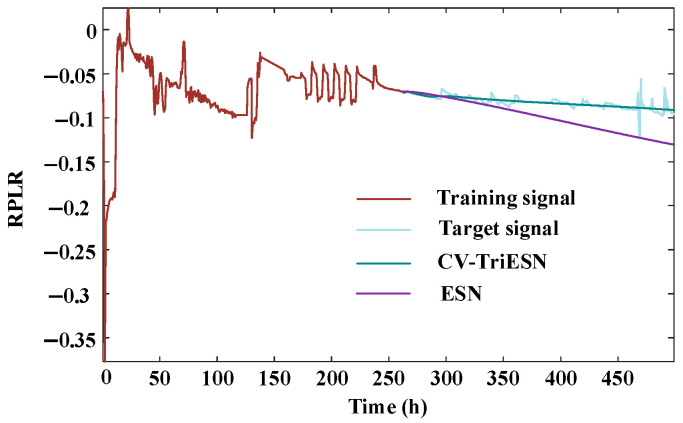
The prediction under experiment 2.

The CV-TriESN model exhibits significantly better performance advantages over the traditional ESN in long-term RPLR forecasting tasks. As shown in Figure 8, over a training period of up to 250 h, the fit of the CV-TriESN’s prediction curve to the target signal is noticeably higher than that of the ESN. The CV-TriESN’s predicted values can dynamically capture the nonlinear decay characteristics of the RPLR, with its error curve showing a smooth downward trend. In contrast, the prediction results of the traditional ESN exhibit a clear accumulation of errors, revealing its limitations in long-term forecasting. Table 2 presents the prediction results of the ESN, LSTM, Transformer, and CV-TriESN over different training durations. The CV-TriESN improves long-term RPLR forecasting accuracy through multi-timescale inputs and reservoir decoupling. The CV-TriESN demonstrates RMSE reductions of 34.41%, 72.63%, and 25.00% at 200 h, 250 h, and 300 h, respectively, and MAPE decreases of 46.31%, 81.39%, and 54.17% compared to the ESN. This improved long-range RPLR forecast accuracy is achieved through multi-timescale feeding signals and reservoir partitioning in the CV-TriESN. In different training stages, the CV-TriESN is superior to the other models in RMSE and MAPE. For example, in the 200 h training stage, the RMSE of the CV-TriESN is 0.00448, which is significantly lower than other methods. In addition, the Transformer model is second, and its error is 5–15% higher than that of the CV-TriESN, while the LSTM model is 10–20% higher than that of Transformer. As a benchmark model, the ESN has the weakest performance, which further proves the superiority and reliability of the CV-TriESN in predicting the aging process of PEMFCs.

### 4.3. Under Dynamic Conditions (Experiment 3)

**Figure 9:**
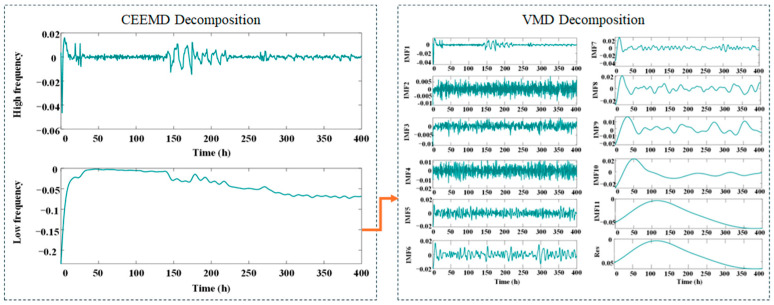
The result of decomposition under experiment 3 by CEEMD-VMD.

**Figure 10:**
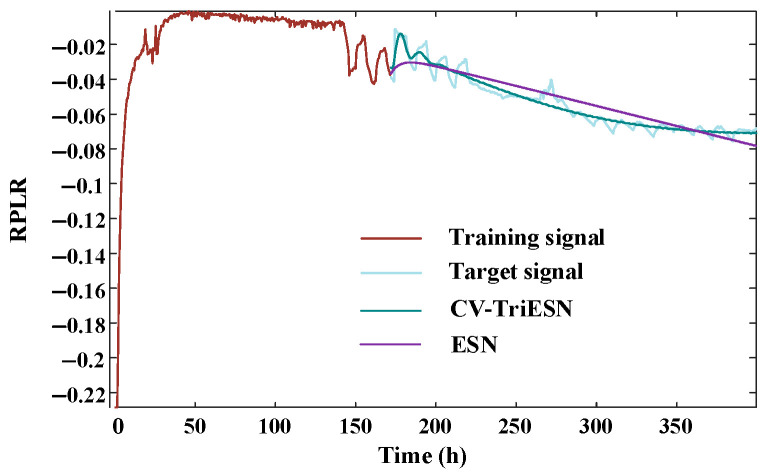
The prediction under experiment 3.

In experiment 3, the proportion of training is set to 43.75%, 50%, 56.25%, and 62.5% of the total length 400 h. The third dataset comprised 400 h of preprocessed measurements, which were systematically divided into four temporal subsets for analysis. The decomposition results obtained through CEEMD-VMD analysis of the aging RPLR signals are illustrated in Figure 9. The CV-TriESN model demonstrates significant performance advantages over the standard ESN architecture in RPLR prediction tasks. As illustrated in Figure 10, after a training period of 170 h, the fit of the CV-TriESN’s prediction curve to the target signal is significantly better than that of the ESN. Specifically, the RPLR trend predicted by the CV-TriESN exhibits strong agreement with empirical measurements. Although the echo state network generates linear forecasts, the CV-TriESN demonstrates a nonlinear trajectory that more accurately follows the anticipated RPLR deterioration profile. Table 3 illustrates the predictive capability comparison of the ESN, LSTM, Transformer, and CV-TriESN models over different training durations. The CV-TriESN model achieves higher accuracy in long-term RPLR predictions by utilizing multi-timescale inputs and employing reservoir decoupling techniques. The CV-TriESN demonstrates RMSE reductions of 27.47%, 18.96%, and 37.05% at 175 h, 200 h, and 225 h, respectively, and MAPE decreases of 19.89%, 26.62%, and 48.99% compared to the ESN. This improved long-term RPLR prediction precision is achieved through multi-timescale inputs and reservoir decoupling in the CV-TriESN. In different training stages, the CV-TriESN is superior to the other models in RMSE and MAPE indicators, especially in the 250 h training stage, its RMSE is only 0.00250, which is significantly lower than other methods. In addition, the performance of the CV-TriESN remained stable at all stages, showing its superiority and reliability in PEMFC aging prediction.

At the stage of training, the standard ESN has an advantage in a short time because it only needs linear regression to fit the output weight. It is very suitable for real-time applications. The CV-TriESN train time is prolonged because of signal decomposition. At the stage of predicting, the time of the TriESN and ESN is similar and has little effect. Also, the CV-TriESN may reach the second level because of adding decomposition time. Though the CV-TriESN is time-consuming due to the process of signal decomposition, life prediction and health status assessment allows second-order delay in terms of real-time monitoring. It could realize real-time monitoring; meanwhile, the prediction accuracy is further improved using the CV-TriESN.

### 4.4. Experimental Verification of Decomposition Methods

The advantages of the CV-TriESN are mainly reflected in three aspects. First, the cross-validation mechanism effectively avoids over-fitting and keeps the model stable in long-term training. Secondly, the variational mode decomposition accurately extracts the signal features and improves the feature utilization rate. Finally, the triplet reservoir structure enhances the expression ability of the model. These characteristics make it particularly suitable for monitoring tasks of complex time-varying systems and provide a reliable technical solution for real-time state assessment. Compared to different decomposition methods, the simulation results are shown in Table 4. The different ways include the TriESN, VMD-TriESN (V-TriESN), CEEMD TriESN (C-TriESN), and CV-TriESN in the condition of experiment 3. Balanced on weighing the calculation cost and accuracy, it could be realized to analyze the experimental results.

Through data analysis, the CV-TriESN showed excellent performance in all the training stages. In the 250 h training phase, the RMSE (0.00250) and MAPE (0.03216%) of the CV-TriESN were significantly lower than those of the other models, which were 15.3% and 13.7% higher than those of the sub-optimal C-TriESN, respectively. Especially in the critical training period of 225–250 h, the CV-TriESN has the greatest performance improvement, indicating that it has better long-term stability.

### 4.5. Estimation of Remaining Useful Life

**Figure 11:**
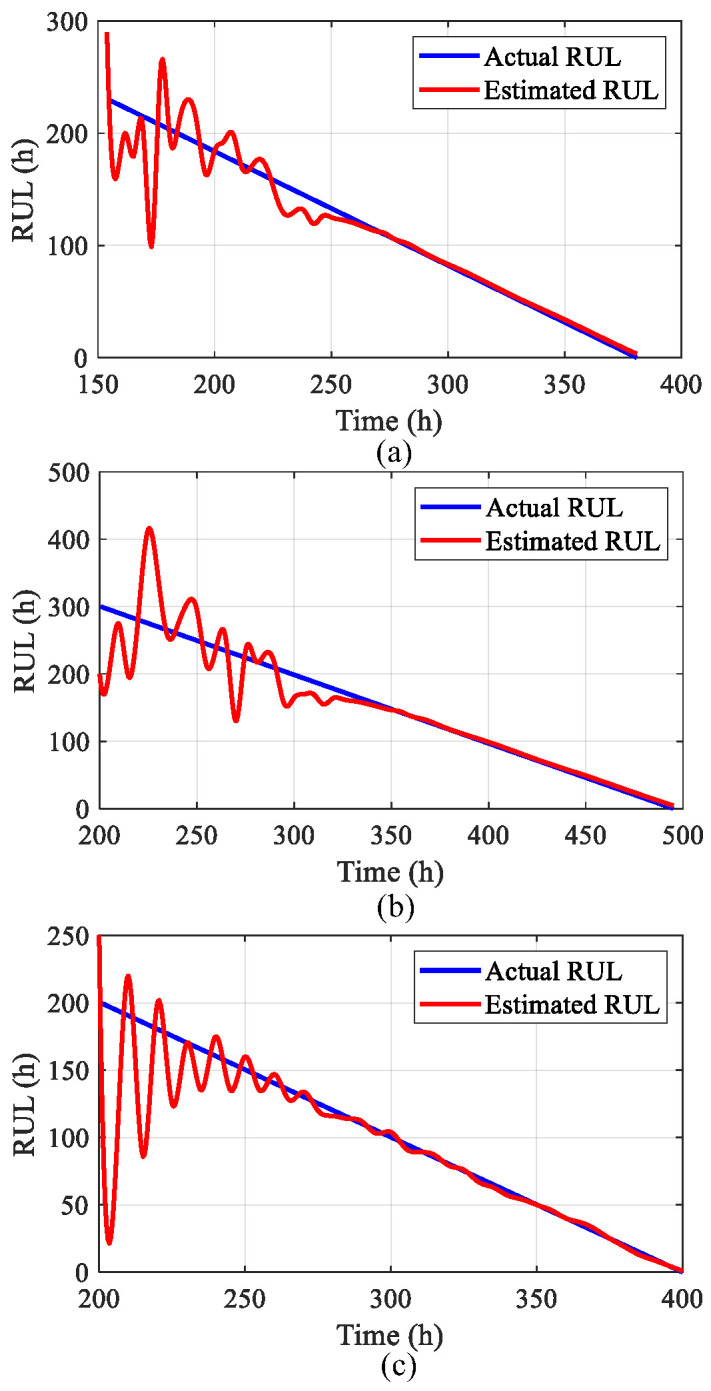
RUL calculation consequence across different test conditions: (**a**) experiment 1; (**b**) experiment 2; (**c**) experiment 3.

This study utilizes RMSE and MAPE to quantitatively assess prediction accuracy, measuring the discrepancy between observed and predicted values throughout the forecasting period. Lower metric values correspond to improved RPLR trend prediction precision. The developed network model detects when predicted RPLR trajectories exceed predefined failure thresholds, enabling the RUL computation. Figure 11 presents the resultant RUL estimation outcomes.

## 5. Conclusions

Accurate RUL prediction for PEMFCs hinges on selecting appropriate degradation indicators and reliably forecasting the degradation path. Throughout the PEMFC system’s degradation process, the ESN is capable of predicting RPLR across multiple time steps. By stacking reservoirs, the forecasting accuracy of the ESN model is significantly enhanced. However, the internal aging of the system involves multi-timescale issues, compounded by the coupling of neurons and sub-reservoirs. Initially, high-frequency noise is filtered out through CEEMD decomposition, and the system’s aging trend is extracted. Subsequently, to refine the low-frequency signal obtained from CEEMD decomposition, it is crucial to employ VMD to enhance the degradation signal’s clarity. The TriESN fortifies feature representation, network sparsity, and generalization capabilities, accelerating the learning of useful information and bolstering prediction accuracy. This research investigates the application of the TriESN for PEMFC aging forecast modeling, with experimental results demonstrating superior prediction accuracy evidenced by minimized RMSE and MAPE values across multiple operating conditions. Building upon these findings, subsequent studies should explore the synergistic integration of real-time estimation approaches with extended prediction horizon methodologies.

## Figures and Tables

**Table 1:** The consequence of experiment 1.

Training Phase (Experiment 1)	RMSE		MAPE	
	ESN	CV-TriESN	ESN	CV-TriESN
200 h	0.00394	0.00377	0.07216	0.06404
250 h	0.00265	0.00222	0.03987	0.03563
300 h	0.00104	0.00109	0.01517	0.01437
350 h	0.00110	0.00097	0.01512	0.01193
**Other Methods**	**LSTM**	**Transformer**	**LSTM**	**Transformer**
200 h	0.00385	0.00365	0.07012	0.06633
250 h	0.00245	0.00230	0.03745	0.03621
300 h	0.00112	0.00105	0.01505	0.01412
350 h	0.00105	0.00099	0.01285	0.01210

**Table 2:** The consequence of experiment 2.

Training Phase (Experiment 2)	RMSE		MAPE	
	ESN	CV-TriESN	ESN	CV-TriESN
200 h	0.00683	0.00448	0.05886	0.03160
250 h	0.01805	0.00494	0.17373	0.03233
300 h	0.00776	0.00582	0.07764	0.03558
350 h	0.00999	0.00881	0.09738	0.08762
**Other Methods**	**LSTM**	**Transformer**	**LSTM**	**Transformer**
200 h	0.00512	0.00475	0.04218	0.03544
250 h	0.00633	0.00562	0.05127	0.04015
300 h	0.00688	0.00605	0.05692	0.04833
350 h	0.00915	0.00895	0.08947	0.08801

**Table 3:** The consequence of experiment 3.

Training Phase (Experiment 3)	RMSE		MAPE	
	ESN	CV-TriESN	ESN	CV-TriESN
175 h	0.00495	0.00359	0.07686	0.06157
200 h	0.00422	0.00342	0.06763	0.04963
225 h	0.00440	0.00277	0.06687	0.03411
250 h	0.00818	0.00250	0.08243	0.03216
**Other Methods**	**LSTM**	**Transformer**	**LSTM**	**Transformer**
175 h	0.00435	0.00382	0.06925	0.06533
200 h	0.00388	0.00365	0.05847	0.05321
225 h	0.00362	0.00315	0.04892	0.04077
250 h	0.00495	0.00380	0.05914	0.04532

**Table 4:** The consequence of different methods (experiment 3).

Training Phase (Experiment 3)	RMSE		MAPE	
	TriESN	V-TriESN	TriESN	V-TriESN
175 h	0.00435	0.00415	0.06844	0.06522
200 h	0.00402	0.00375	0.05589	0.05541
225 h	0.00385	0.00325	0.04026	0.04233
250 h	0.00312	0.00380	0.03805	0.04512
**Other Methods**	**C-TriESN**	**CV-TriESN**	**C-TriESN**	**CV-TriESN**
175 h	0.00392	0.00359	0.06015	0.06157
200 h	0.00365	0.00342	0.05277	0.04963
225 h	0.00310	0.00277	0.03944	0.03411
250 h	0.00295	0.00250	0.03728	0.03216

## Data Availability

This study’s raw sequence data is archived in the Fuel Cell Laboratory (FCLAB).
